# Determination of the Predictive Value of Serum Bilirubin in Patients with Ischemic Stroke: A Prospective Descriptive Analytical Study

**DOI:** 10.15171/apb.2018.080

**Published:** 2018-11-29

**Authors:** Elnaz Sagheb Asl, Aliakbar Taheraghdam, Farzad Rahmani, Reza Javadrashid, Samad Eslam Jamal Golzari, Neda Ghaemian, Yalda Sadeghpour, Robab Mehdizadeh Esfanjani, Hassan Soleimanpour

**Affiliations:** ^1^Emergency Medicine Research Team, Tabriz University of Medical Sciences, Tabriz, Iran.; ^2^Neurosciences Research Center, Tabriz University of Medical Sciences, Tabriz, Iran.; ^3^Department of Radiology, Imam Reza Teaching Center, Tabriz University of Medical Sciences, Tabriz, Iran.; ^4^Research Center for Evidence Based Medicine, Tabriz University of Medical Sciences, Tabriz, Iran.; ^5^Aging Research Institute, Tabriz University of Medical Sciences, Tabriz, Iran.; ^6^Students’ Research Committee, Tabriz University of Medical Sciences, Tabriz, Iran.

**Keywords:** Bilirubin, Stroke, Emergency Department

## Abstract

***Purpose:*** In all types of ischemic stroke, especially in the acute phase, excessive oxidative stress causes structural and functional damage to the brain. This may play a major role in the pathophysiology of the brain damage. Higher serum levels of bilirubin have therapeutic effects in oxidative stress-induced stroke. Nevertheless, role of increased serum levels of bilirubin in the acute phase of ischemic stroke is ccontroversial.

***Methods:*** This study was a cross-sectional prospective descriptive study conducted in the Emergency Department (ED) of Imam Reza hospital, Tabriz University of Medical Sciences, Tabriz, Iran, throughout six months. 275 ischemic stroke patients were evaluated based on their brain CT scan infarct size, NIHSS, MRS, and serum levels of bilirubin. Later, data were analyzed using SPSS software.

***Results:*** Results: Total, direct and indirect bilirubin levels were significantly higher in expired patients (p < 0.0001). Total (p< 0.0001), direct (p< 0.0001) and indirect (p< 0.0001) bilirubin levels, NIHSS score (p< 0.0001), and ischemic area (p< 0.0001) significantly predicted the outcome in these patients.

***Conclusion:*** Total, direct and indirect bilirubin levels was significantly associated with mortality in the acute phase of ischemic stroke patients.

## Introduction

Stroke is known as a clinical syndrome with focal neurologic defects which last longer than 24 hours. It is the first cause of disability in both developed and developing countries. In most studies, stroke has been introduced as the second most common cause of death.^1-5^ On the other hand, not only is the prevalence of stroke increasing but also the age of stroke is sharply decreasing in developed and developing countries.^6-8^ In all types of ischemic stroke, excessive oxidative stress causes structural and functional damage to the brain, especially throughout the acute phase; this may play a major role in the pathophysiology of brain damage.^9-12^ Bilirubin is the ultimate product of heme metabolism and, when accumulated with high concentrations in tissues, it becomes highly toxic. However, bilirubin is known to be of very potent antioxidant properties.^13^ High serum levels of bilirubin have therapeutic effects in diseases caused by oxidative stress.^7^ However, these changes and their role in the acute phase of ischemic stroke are less known.^9^In a study, it has been shown that with an increase in the bilirubin levels, the risk of stroke is reduced; the correlation, nevertheless, was not found to be significant in either hemorrhagic stroke or women patients. This study eventually concluded that bilirubin may have a protective effect against the risk of stroke in men.^6^ Interestingly, patients experiencing stroke had lower total bilirubin levels.^14^ In ischemic stroke, numerous changes occur in the liver enzymes and bilirubin levels;^15^ liver enzymes and CRP increase during the first week of ischemic stroke, but the levels of conjugated and non-conjugated bilirubin, erythrocyte and hemoglobin concentration decreases.^16^ Nevertheless, serum levels of alkaline phosphatase and direct bilirubin and bile duct diameter remain constant.^4^Increased levels of direct and total bilirubin serum levels after ischemic stroke have also been introduced as indicators of stroke severity.^9^ In a study by Arsalan et al., high serum bilirubin levels increased the severity of the stroke, the length of hospitalization, and poor prognosis in patients.^1^ Considering the existing controversy in various studies, the high prevalence of stroke and the importance of any probable predictive factor, we examined the role of bilirubin in predicting the outcome of patients with ischemic stroke.

## Materials and Methods

This study was approved by the Ethics Committee of Tabriz University of Medical Sciences (TUOMS) and registered under the Code Number of 5/4/12427. Informed written consents were obtained from all parents. 260 patients with ischemic stroke were enrolled and examined daily in Imam Reza Emergency Medical Center during the 6 months. Patients who suffered at least one of the three symptoms of hemiparesia, unilateral paralysis of the face or dysarthria were included according to the Cincinnati pre-hospital stroke scale. After initial measures and brain CT scan were taken, information on patients with acute ischemic stroke was collected. This information included risk factors for coronary heart disease, myocardial infarction and atrial fibrillation, total, indirect and indirect bilirubin, and history of hypertension, diabetes, smoking and alcohol consumption. Serum total bilirubin was measured in the emergency room lab (using Pars AzmoonKit93004). Exclusion criteria included patients with Gilbert's or active liver-bile duct disease. Hypertension was defined as having a history of hypertension or BP≥140/90 in two measurements in the acute stroke phase. Diabetes includes all patients who have a medical report or take oral agent medications or insulin. Hyperlipidemia will also be diagnosed if documented in a medical history. Smokers are known to have a history of smoking over the past 5 years. The neurological function and severity of the stroke were measured using the National Institutes of Health Stroke Scale (NIHSS), a 15-item tool for measuring the severity of brain damage indicating the level of consciousness-vision-sense-movement, lingual function and perception in patients. The range is between 0 and 24. Higher rating indicates higher severity. This indicator was measured on admission and discharge. Scores between 1-4, 5-15 and 16-20 indicate mild, moderate and severe stroke, respectively. Functional outcomes of the patients were measured using the MRS (Modified Rankin Scale), indicating the level of activity and performance compared with the time before the stroke with a score of 0 to 5; zero indicates asymptomatic and 5 indicates severe disability. The data were analyzed using SPSS software. Results were reported as mean and Frequency (%). To test the normal distribution of variables we used the Kolmogrov-Smirnof test. Chi-square test (if necessary Fisher exact test) was used to determine the relationship between qualitative variables. To compare quantitative variables between two groups we used T-student test. Receiver Operating Characteristic (ROC) Curve Analysis was used to determine the cutoff points of bilirubin levels for predicting the outcome of patients with stroke. The values ​​of P less than 0.05 was considered statistically significant.

## Results

**Figure 1:**
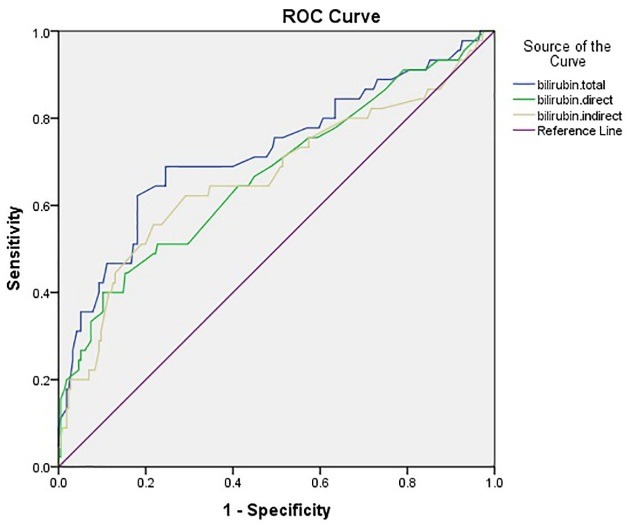
ROC Curves for sensitivity and specificity of total, direct and indirect bilirubin for diagnosis of patients’ outcome

**Figure 2:**
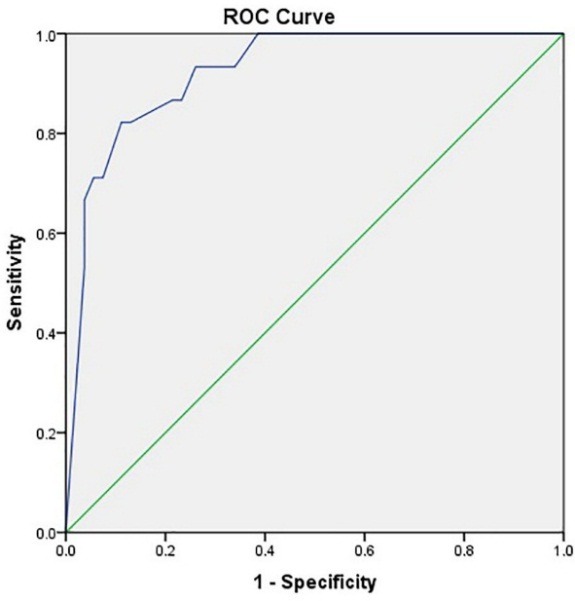
ROC Curve for sensitivity and specificity of NIHSS for diagnosis of patients’ outcome

**Figure 3:**
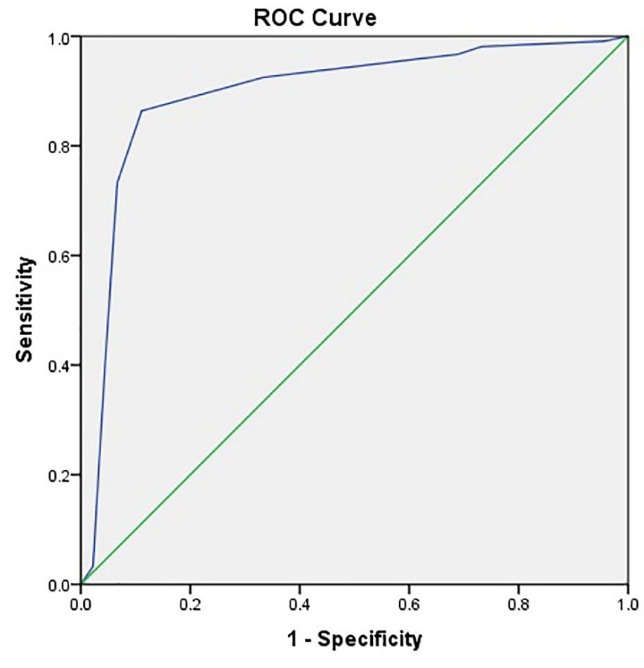
ROC Curve for sensitivity and specificity of Ischemic area for diagnosis of patients’ outcome

Of the 260 patients, 146 (56%) were male and 114 were female (44%). Of all patients, 215 (82.7%) were discharged and 45 (17.3%) died (P=0.4); 212 (81.5%) were admitted to the neurology department, and 48 (17.5%) were admitted to the ICU. Most of the patients in the neurology department (97%) were discharged, while most ICU patients (84%) died (P<0.0001). Forty eight patients (18%) underwent mechanical ventilation. Results showed that mortality was significantly higher among those requiring mechanical ventilation than other patients (P<0.0001). Facial drop was evaluated in 140 patients, of which 54 (38%) were normal and 87 (62%) were abnormal. Tables 1 and 2 discuss the prevalence of risk factors in patients and their impact on the outcome of patients. The cutoff points of the total bilirubin level (0.99 with the sensitivity 0.69 and specificity 0.68), direct (0.29 with the sensitivity 0.64 and specificity 0.59) and indirect (0.64 with the sensitivity 0.64 and specificity 0.65) bilirubin levels were evaluated in predicting the outcome of the patients by the Rock's curves which means that larger values of the cutoff points indicate stronger evidence for die (Figure 1). The areas under the curves were 0.72, 0.67 and 0.67, respectively. In Figures 2 and 3, the NIHSS score and the ischemic area have been shown, respectively. The cutoff points of the NIHSS (16.5 with the sensitivity 0.87 and specificity 0.87) and ischemic area (6.5 (for discharge) with the sensitivity 0.86 and specificity 0.89) were calculated for predicting the outcome of patients. The most reliable predictors were the total bilirubin, the NIHSS score and the ischemic area with the level of 0.72, (0.92) and (0.89) area under the curve, respectively.

## Discussion

The purpose of this study was to determine the predictive value of bilirubin levels in the outcome of patients with ischemic stroke. In this study, 260 patients with ischemic stroke referred to Imam Reza Tabriz Hospital were collected and reviewed. According to the results, 146 (56%) of patients was males and 114 females (44%), which were men more than the women. Similarly, in the Kimm et al. study,^10^ the number of stroke cases in men was higher than women, which could indicate a higher incidence of stroke among men. The outcome of the disease among the patients showed that 215 (82.7%) of the patients were discharged, while 45 (17.3%) died. Investigating the sensitivity and specificity of the total, direct and indirect total bilirubin levels in predicting the outcomes of patients by the Rock Curves revealed that all three variables were significantly predictive of the outcome of the patients. Consequently, the results of this study showed that increased levels of bilirubin intensifies the severity of stroke and consequently increases mortality among patients. Similar results were reported in the study by Luo et al. which showed that the direct and total bilirubin levels after ischemic stroke were higher than the control group, indicating stroke severity.^12^ Pinedu et al. also found that high direct bilirubin levels are associated with higher severity of stroke and poor outcomes during discharge; nevertheless, there is no direct relation between direct bilirubin and outcome during admission.^17^ Moreover, there was no correlation between total bilirubin and severity of stroke or outcome of the disease in this study. Arsalan and colleagues also reported that high levels of serum bilirubin increased stroke severity, prolonged hospitalization and poor prognosis in patients.^1^ In contrast, some studies have reported decrease in the levels of indirect bilirubin in the acute phase of ischemic stroke and lack of change in the levels of direct bilirubin which might be related to the reduction in the production and destruction of red blood cells and heme cycle. Acute phase of stroke triggers inflammation in the body. This would disrupt the heme cycle and reduce the destruction and production of red blood cells, which also reduces the production of indirect bilirubin in the acute phase of ischemic stroke. On the other hand, the presence of systemic inflammation increases the level of direct bilirubin as an inflammatory factor. This increase is countered by the decrease in the level of bilirubin resulting from the disruption of the heme cycle, and causes no significant change in the level of direct bilirubin.^4^ However, this study did not consider the outcome of patients and the severity of the ischemic stroke in the study, and it only examined the changes in bilirubin levels during the acute phase. In fact, the nature of the two studies is different which might explain the difference in results to some extends. Of the total patients, 212 (81.5%) and 48 (17.5%) were admitted to the neurology department and ICU, respectively. Investigating the outcomes of patients according to the status of admission showed that most of the patients (97%) were discharged in the neurology department, while most (84%) died in the ICU. Therefore, the condition of admission can also be a good predictor in determining the outcome of patients. Frequency and incidence of complications such as facial drops, arm drift, mechanical ventilation, ischemic heart disease, dysarthria, Calcium Channel Blockers or aspirin usage, hypoglycemic agents, hypertension, hyperlipidemia, dysrhythmia, LBBB, RBBB and AV Block and risk factor such as diabetes, alcohol consumption, and smoking among patients were examined by gender and also by outcome. The results of the study showed that gender composition does not follow a specific pattern, and there is a significant difference between the groups in cases of alcohol (*P=0.03*) and smoking (*P<0.0001*). But the mortality rate in patients with ischemic heart disease, hypertension, diabetes, cardiac dysrhythmia, mechanical ventilation, facial drops, arm drift, dysarthria, ACEI or ASA usage, AF, hypoglycemic agent, hyperlipidemia was significantly higher. Therefore, each of these can be considered as predictors of the severity or poor outcome in ischemic stroke patients. Studied variables including levels of total, direct and indirect bilirubin, NIHSS score and ischemic area are significant predictors of outcome in patients.

**Table 1:** Demographic Characteristics and clinical Findings of patients

-			**Sex**		**Outcome**					**sex**		**outcome**	
			**male**	**female**	**discharge**	**die**				**male**	**female**	**Discharge**	**die**
**Dysarthria**	No	29	15	14	29	0	Hyperlipidemia	No	222	124	98	190	32
	Yes	172	98	74	135	37		Yes	39	22	17	26	13
	p-value		0.59		<0.0001			p-value		0.94		0.01	
**ACEI**	No	133	72	60	121	12	Smoking	No	226	118	108	186	39
	Yes	128	74	54	95	33		Yes	35	28	7	30	6
	p-value		0.34		<0.0001			p-value		<0.0001		0.7	
**Ca.Channel.Blocker**	No	217	122	95	184	34	ACS	No	193	109	84	169	24
	Yes	43	44	19	32	11		Yes	67	36	31	46	21
	p-value		0.96		0.12			p-value		0.7		<0.0001	
**ASA**	No	161	90	60	137	13	Dysrhythmia	No	194	103	91	171	21
	Yes	99	56	54	79	32		Yes	66	42	24	44	23
	p-value		0.14		<0.0001			p-value		0.15		<0.0001	
**Hypoglycemic. Agent**	No	150	90	71	141	20	AF	No	211	116	95	185	26
	Yes	110	56	43	75	25		Yes	49	29	20	30	19
	p-value		0.91		0.01			p-value		0.61		<0.0001	
**Alcohol**	No	245	133	112	205	40	LBBB	No	247	137	110	207	39
	Yes	15	13	2	11	5		yes	13	9	4	9	5
	p-value		0.03		0.15			p-value		0.24		0.06	
**Hypertension**	No	92	55	37	88	4	RBBB	No	251	142	109	207	44
	Yes	169	91	78	127	42		Yes	9	3	6	8	1
	p-value		0.35		<0.0001			p-value		0.19		1.0	
**DM**	No	149	89	60	130	19	AV.Block	No	258	144	114	213	45
	Yes	112	57	55	86	26		Yes	3	2	1	3	0
	p-value		0.15		0.03			p-value		NA		NA	

**Table 2:** Vital signs, duration of time a patient is on mechanical ventilation and laboratory findings of patients

-	**Outcome**	**N**	**Mean**	**Std-dv**	**p-value**
**Duration of time a patient is on mechanical ventilation**	Discharge	2	12.5	3.5	0.7
	Die	35	9.4	11.3	-
**MAP**	Discharge	215	85.3	16.1	<0.0001
	Die	45	95.2	23.3	-
**HR**	Discharge	215	96.8	19.5	<0.0001
	Die	45	123.8	28.3	-
**Total bilirubin**	Discharge	216	.89	.41	<0.0001
	Die	45	1.42	.74	-
**Direct bilirubin**	Discharge	216	.30	.18	0.001
	Die	45	.53	.42	-
**Indirect bilirubin**	Discharge	216	.60	.28	0.001
	Die	45	.87	.51	-
**BS**	Discharge	216	144.7	76.7	0.91
	Die	45	143.5	52.0	-
**Cr**	Discharge	215	1.08	.76	0.38
	Die	45	1.20	1.29	-
**Na**	Discharge	213	141.9	4.4	0.14
	Die	45	140.9	3.4	-
**K**	Discharge	214	4.2	.44	0.81
	Die	45	4.2	.33	-
**Platelet**	Discharge	215	291641	328058	0.58
	Die	44	321340	346480	-

## Conclusion

The current study demonstrated that increased levels of bilirubin is associated with the severity of stroke and ischemic area which in turn leads to an increased mortality rate in these patients.

## Acknowledgments

We are grateful to all who participated in this study. Our deep appreciation goes to data collectors, supervisors, and administrative staff of the Emergency Medicine and Neurology departments in Imam Reza Hospital, Tabriz, Iran. This article was written based on dataset of Elnaz Sagheb Asl’s Medical Specialty degree thesis entitled, “**Determination of the predictive value of serum bilirubin in patients with ischemic stroke: A Prospective descriptive analytical study**”. This study was registered in Tabriz University of Medical Sciences (No: 94/3-6/12). We would also like to thank Dr. Rassul Hassanpour, MD for his help during our research.

This article was supported by Neurosciences Research Center, Tabriz University of Medical Sciences, Daneshgah Street, Tabriz, 51664, Iran. Special thanks to the research vice chancellor of Tabriz University of Medical Sciences for all the material and financial support for this study.

## Ethical Issues

An institutional ethical approval was obtained for this work from Tabriz University of Medical Sciences and a signed written informed consent form was obtained from patient.

## Conflict of Interest

The author(s) declare that they have no competing interest.
